# Impact of Biannual Mass Azithromycin Treatment on Enteropathogen Carriage in Children <5 Years Old in Niger

**DOI:** 10.1093/cid/ciab1046

**Published:** 2022-01-10

**Authors:** James A Platts-Mills, Elias G Ayoub, Jixian Zhang, Elizabeth T Rogawski McQuade, Ahmed M Arzika, Ramatou Maliki, Amza Abdou, Jeremy D Keenan, Thomas M Lietman, Jie Liu, Eric R Houpt

**Affiliations:** Division of Infectious Diseases & International Health, University of Virginia, Charlottesville, Virginia, USA; University of Virginia School of Medicine, University of Virginia, Charlottesville, Virginia, USA; Division of Infectious Diseases & International Health, University of Virginia, Charlottesville, Virginia, USA; Department of Public Health Sciences, University of Virginia, Charlottesville, Virginia, USA; Centre de Recherche et d’Intervention en Santé Publique, Niamey, Niger; Centre de Recherche et d’Intervention en Santé Publique, Niamey, Niger; Programme National de la Santé Oculaire, Ministere de la Santé Publique, Niamey, Nigerand; Francis I Proctor Foundation, University of California, San Francisco, California, USA; Francis I Proctor Foundation, University of California, San Francisco, California, USA; Division of Infectious Diseases & International Health, University of Virginia, Charlottesville, Virginia, USA; Division of Infectious Diseases & International Health, University of Virginia, Charlottesville, Virginia, USA

**Keywords:** Azithromycin, enteropathogens, *Shigella*

## Abstract

We analyzed samples obtained at baseline and 24 months in a mass azithromycin administration trial in Niger using quantitative polymerase chain reaction. In villages randomized to azithromycin, *Shigella* was the only pathogen reduced at 24 months (prevalence ratio, 0.36 [95% confidence interval: .17–.79]; difference in log quantity, −.42 [−.75 to −.10]).

The MORDOR I trial was a cluster-randomized placebo-controlled trial demonstrating that biannual mass azithromycin administration reduced the all-cause mortality rate in children <5 years old in Niger, Malawi, and Tanzania [1]. Analysis of verbal autopsies from communities enrolled in Niger found reductions in malaria, dysentery, meningitis, and pneumonia deaths [2], and most deaths in children <5 years old in sub-Saharan Africa are attributed to infections [3]. Mass azithromycin administration could reduce transmission of specific microbial etiologies.

Understanding the effects of azithromycin in the MORDOR I trial requires analysis of objective microbiologic end points. Ninety-one communities from the 3 countries included in MORDOR I were included in a parallel trial with the same intervention and specimen collection [1]. We have previously used quantitative polymerase chain reaction (PCR) testing to identify enteric pathogens in similar settings [4]. In the current study, we tested rectal swab samples obtained from children aged 1–59 months in 30 of these communities to evaluate the impact on carriage of enteric pathogens.

## MATERIALS AND METHODS

### Study Design and Sample Collection

The MORDOR trial has been described elsewhere [1]. For the microbiologic substudy, 30 communities from Niger were randomized in a 1:1 ratio to biannual administration of azithromycin or placebo. Rectal samples were obtained from 10 randomly selected children aged 1−59 months per community at both baseline and 24 months and stored in DNA/RNA Shield (Zymo Research). Sampling did not take into consideration whether the child currently or recently had diarrhea. These samples were previously analyzed by Doan et al [5] using metagenomic RNA sequencing.

### Sample Testing

All samples were placed on ice, stored at −20°C in Niger, and then shipped on dry ice and stored at −80°C. DNA and RNA were extracted with Norgen DNA and RNA isolation kits at the University of California, San Francisco. We tested available samples with quantitative PCR assays for 29 enteropathogens. All procedures, including assay validation, quantitative PCR setup, and quality control, have been described elsewhere [4, 6]. We included 1 no-template amplification control per 10 cards to monitor for laboratory contamination.

### Statistical Analysis

We modeled the association between intervention arm and pathogen quantity and detection, both at baseline and at 24-month follow-up. For pathogen quantity, we transformed cycle threshold (Ct) values to a log_10_ scale and set pathogen quantities for stool samples in which the pathogen was not detected (Ct, ≥35) at half the limit of detection. We used generalized estimating equations to fit a linear model to estimate the association between azithromycin arm and pathogen quantity, with village as the cluster variable.

This modeling strategy performed equally well as a parametric g-computation approach with a 2-part model, despite model misspecification due to the zero-inflated semicontinuous outcome data [7]. The models for the follow-up samples were adjusted for the village-level mean log quantity of that pathogen at baseline to adjust for baseline differences in pathogens between villages. For pathogen detection, we estimated prevalence ratios using log-binomial regression and prevalence differences using log-linear regression. A Ct of 30 was used as the detection cutoff to exclude detections of low pathogen quantities. Finally, we estimated the association between the azithromycin arm and pathogen prevalence and quantity at follow-up, stratified by age. All pathogens with a baseline prevalence of >2% were included in the models. Analyses were performed using R software (version 4.0.2; R Foundation for Statistical Computing).

## RESULTS

Of 600 rectal swab samples, 540 (90.0%) had sufficient sample for testing, including 263 at baseline (128 from villages randomized to placebo and 135 from villages randomized to azithromycin) and 277 at 24 months (140 from placebo and 137 from azithromycin villages). Five pathogens had a baseline prevalence >2%: enteroaggregative *Escherichia coli* (21.7%), *Shigella* (14.1%), *Campylobacter jejuni* or *Campylobacter coli* (12.9%), enterotoxigenic *E. coli* (11.8%), and typical enteropathogenic *E. coli* (3.8%). The prevalence of *Shigella* was high, even in infants, with *Shigella* detected in 8 of 60 samples (13.3%) from infants aged 1–11 months and 55 of 343 (16.0%) from children aged 12–59 months, collected either at baseline or from villages receiving placebo at 24-month follow-up.

**Figure 1.**
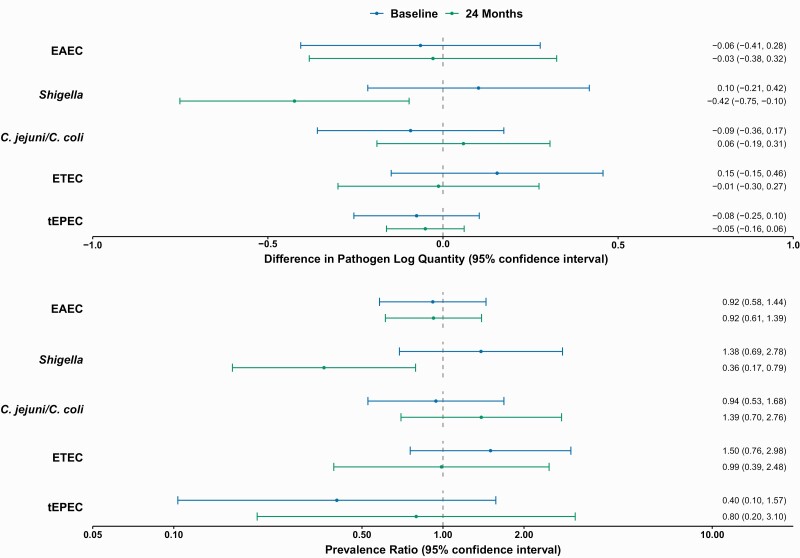
Impact of biannual mass azithromycin treatment on enteric pathogen carriage in the MORDOR I study of children <5 years of age in Niger. All pathogens with a baseline prevalence >2% were included, and 24 month estimates for each pathogen are adjusted for the village-level mean log quantity of that pathogen at baseline. *A,* Difference in pathogen log quantity between treatment arms at baseline and 24 months. *B,* Prevalence ratios (ratio of azithromycin arm to placebo arm) at baseline and 24 months. Abbreviations: *C. coli, Campylobacter coli*; *C. jejuni, Campylobacter jejuni*; CI, confidence interval; EAEC, enteroaggregative *Escherichia coli*; ETEC, enterotoxigenic *E. coli*; tEPEC, typical enteropathogenic *E. coli*.

At baseline, there were no differences in pathogen quantity by arm. In villages receiving azithromycin, *Shigella* log quantity was lower after 24 months than in villages receiving placebo (difference in log quantity, −0.42 [95% confidence interval (CI): −.75 to −.10]) (Figure 1A). This reduction was apparent across ages groups, with a reduction demonstrated in both infants (difference, −0.67; [95% CI: −1.35 to .01]) and older children (−0.38 [−.72 to −.04]). Similarly, there was no baseline difference in pathogen prevalence between arms, while at 24 months, the prevalence of *Shigella* in samples from villages randomized to azithromycin was 64% lower than in samples from villages receiving placebo (prevalence ratio, 0.36; 95% CI: .17–.79) (Figure 1B), corresponding to an absolute prevalence difference of −11.1% (−19.4% to −2.8%).

The point estimate of the prevalence ratio was 64% in both age groups (prevalence ratio, 0.36 [95% CI: .09–1.42] for children aged 1–11 months and 0.36 [.17–.79] for those aged 12–59 months). Within the age categories defined in the MORDOR trial, the prevalence of *Shigella* at 24 months for villages receiving placebo versus those receiving azithromycin was as follows: age 1–5 months, 3 of 10 (30%) versus 0 of 12 (0%), respectively; age 6–11 months, 2 of 13 (15.4%) versus 2 of 9 (22.2%); age 12–23 months, 8 of 29 (27.6%) versus 4 of 30 (13.3%); and age 24–59 months, 13 of 88 (14.8%) versus 4 of 86 (4.7%). No other differences in pathogen quantity or prevalence were seen.

## DISCUSSION

This analysis from the Niger cohort of MORDOR I showed a substantial reduction in *Shigella* quantity and prevalence among villages that received biannual azithromycin compared with placebo. These findings have implications for interventions to reduce *Shigella* infection, are consistent with the reduction in dysentery by verbal autopsy in Niger, and may partly explain the mortality benefit observed in the original study.

This reduction in *Shigella* carriage is plausible, because azithromycin is effective at reducing *Shigella* in stool samples [8]. Humans are the only natural hosts of *Shigella*, transmission is fecal-oral with a low infecting dose, and postdiarrheal shedding can be prolonged [9]. While several bacterial enteropathogens are susceptible to azithromycin, restriction to human hosts distinguishes *Shigella* from other pathogens of high prevalence in these settings, including diarrheagenic *E. coli* and most *Campylobacter* species. This host restriction should make *Shigella* particularly susceptible to a mass drug administration program, because the presence of a nonhuman reservoir is a fundamental limitation [10].

These findings suggest that a reduction in *Shigella* infections could have been partly responsible for the mortality benefit seen in MORDOR I. The benefit was most pronounced in Niger and increased over the course of the intervention, consistent with interruption of pathogen transmission [1]. Verbal autopsies from the Niger cohort found diarrhea to be the third most common cause of death and identified a 35% reduction in dysentery in villages randomized to azithromycin [2]. Previous work has revealed significant underdetection of *Shigella* using culture, and the application of quantitative molecular diagnostics have implicated *Shigella* as a major cause of watery diarrhea as well as the primary cause of dysentery [4].

The largest mortality rate reduction in MORDOR I was seen in infants [1]. *Shigella* is thought to be relatively uncommon in infants, with prevalence increasing in the second year of life [4]. However, *Shigella* was identified as a common cause of moderate to severe diarrhea in infants enrolled in a rotavirus clinical trial conducted in Niger [11]. In addition, the high baseline prevalence of *Shigella* observed in infants in this study suggests a high force of infection. In a prior multisite cohort study, the prevalence of *Shigella* by the same diagnostic in nondiarrheal stool samples from infants 0–11 months of age was only 7.2% in Tanzania, and all other sites in the study had a prevalence <3% [4]. Thus, the absolute reduction in *Shigella* carriage observed in the current study was high and, if consistent with a reduction in clinical shigellosis, may have contributed to the observed mortality benefit.

Several limitations should be noted. First, the sample size was powered for an analysis of microbiota composition, and smaller effects or effects on less common pathogens may therefore have been missed [5]. Second, a previous analysis of these samples using metagenomic RNA sequencing did not identify a reduction in *Shigella* [5]. The high degree of similarity between *Shigella* and *E. coli* may also have limited classification to *Shigella* spp. in comparison with the targeted amplification of a differentiating gene [12], or targeting RNA from rectal swab samples may have reduced sensitivity [6]. Third, we could not evaluate the reduction in relative abundance of *Campylobacter upsaliensis* identified in the aforementioned study because we did not include an assay for that species [5]. Finally, the target used for *Shigella*, the invasion plasmid antigen H gene sequence (ipaH), is conserved between *Shigella* and enteroinvasive *E. coli*. However, the latter is relatively rare, and this target is frequently used for detection of *Shigella* [4, 13, 14]

In summary, we found a significant reduction in *Shigella* carriage from a high baseline among children 1–59 months of age in villages that received biannual azithromycin instead of placebo. Together with reports demonstrating a substantial decrease in dysentery incidence, these findings suggest that a reduction in *Shigella* infections may have contributed to the observed reduction in mortality rate.

## Notes

***Financial support*.** This work was supported by the Bill & Melinda Gates Foundation (grant OPP1179069 to E. R. H.).
